# Leptomeningeal disease in neurosurgical brain metastases patients: A systematic review and meta-analysis

**DOI:** 10.1093/noajnl/vdab162

**Published:** 2021-11-10

**Authors:** Ishaan Ashwini Tewarie, Charissa A C Jessurun, Alexander F C Hulsbergen, Timothy R Smith, Rania A Mekary, Marike L D Broekman

**Affiliations:** 1 Department of Neurosurgery, Computational Neuroscience Outcomes Center (CNOC), Brigham and Women’s Hospital, Harvard Medical School, Boston, Massachusetts, USA; 2 Department of Neurosurgery, Leiden University Medical Center, Leiden, Zuid-Holland, the Netherlands; 3 Department of Neurosurgery, Haaglanden Medical Center, The Hague, Zuid-Holland, the Netherlands; 4 Department of Pharmaceutical Business and Administrative Sciences, School of Pharmacy, Massachusetts College of Pharmacy and Health Sciences, Boston, Massachusetts, USA; 5 Department of Neurology, Massachusetts General Hospital, Harvard Medical School, Boston, Massachusetts, USA

**Keywords:** brain metastases, leptomeningeal disease, predictive factors, resection

## Abstract

**Background:**

Leptomeningeal disease (LMD) is a complication distinguished by progression of metastatic disease into the leptomeninges and subsequent spread via cerebrospinal fluid (CSF). Although treatments for LMD exist, it is considered fatal with a median survival of 2–4 months. A broader overview of the risk factors that increase the brain metastasis (BM) patient's risk of LMD is needed. This meta-analysis aimed to systematically review and quantitatively assess risk factors for LMD after surgical resection for BM.

**Methods:**

A systematic literature search was performed on 7 May 2021. Pooled effect sizes were calculated using a random-effects model for variables reported by three or more studies.

**Results:**

Among 503 studies, thirteen studies met the inclusion criteria with a total surgical sample size of 2105 patients, of which 386 patients developed LMD. The median incidence of LMD across included studies was 16.1%. Eighteen unique risk factors were reported as significantly associated with LMD occurrence, including but not limited to: larger tumor size, infratentorial BM location, proximity of BM to cerebrospinal fluid spaces, ventricle violation during surgery, subtotal or piecemeal resection, and postoperative stereotactic radiosurgery. Pooled results demonstrated that breast cancer as the primary tumor location (HR = 2.73, 95% CI: 2.12–3.52) and multiple BMs (HR = 1.37, 95% CI: 1.18–1.58) were significantly associated with a higher risk of LMD occurrence.

**Conclusion:**

Breast cancer origin and multiple BMs increase the risk of LMD occurrence after neurosurgery. Several other risk factors which might play a role in LMD development were also identified.

**Importance of the Study**

Leptomeningeal disease (LMD) is an intractable neuro-oncological complication that is often considered fatal, with a median survival of 2–4 months. The hypothesis is that a relationship exists between LMD and resection due to disruption of anatomical borders, tumor spillage, and thus CSF contamination. Nonetheless, resection has become a cornerstone for treating newly diagnosed brain metastases (BMs). Studies reporting on LMD risk in BM patients were, however, limited in sample size and number of surgically treated patients. This meta-analysis quantitatively assessed what risk factors in surgically treated BM patients were significantly associated with LMD risks and shed light on potential risk factors that are thought to influence LMD occurrence but did not. These results could inform clinicians in deciding what a high-risk profile is for LMD occurrence and initiate future research initiatives to understand LMD occurrence further.

The prognosis of newly diagnosed brain metastasis (BM) patients has improved significantly due to systemic therapies and primary tumor control.^1^ Adequate treatment of BMs has become increasingly fundamental for these patients' survival and quality of life. Patients with extracranial tumor control and a large, solitary BM benefit most from surgical resection with adjuvant radiation therapy, such as stereotactic radiosurgery (SRS).^2^

Although resection has become a cornerstone for treating newly diagnosed BMs, studies have suggested that this treatment is associated with a higher risk for developing leptomeningeal disease (LMD).^3–5^ LMD is defined as metastatic disease progression into the leptomeninges and cerebrospinal fluid (CSF). Hypothetically, the relation between LMD and surgical resection could exist due to the disruption of anatomical borders in brain tissue and surgical spillage of tumor cells, resulting in CSF contamination. Though treatments for LMD exist, the prognosis is abysmal, with a median survival of 2–4 months.^6–8^

Current literature suggests that the LMD risk may vary by BM location, the origin of the primary tumor, treatment modality, and other factors.^4,9,10^ However, most of these studies were limited in sample size and number of surgically treated patients, and a comprehensive overview of risk factors for LMD after neurosurgery is currently lacking.

Therefore, the present study aims to perform a systematic review and meta-analysis of the current literature to summarize risk factors for LMD in BM patients who underwent neurosurgical resection.

## Material and Methods

### Study Design and Search Strategy

A systematic literature search was performed in PubMed, Embase, Web of Science, Cochrane, Academic Search Premier, and PsycINFO according to the Preferred Reporting Items for Systematic Reviews and Meta-Analyses (PRISMA) guidelines on 7 May 2021 (Supplementary Material S1). References of included studies were checked to identify additional relevant publications. Study screening and data extraction were conducted by two independent reviewers (IT and CJ). In case of disagreement, a third reviewer (AH) was consulted.

### In- and Exclusion Criteria

Studies were included if they 1) were randomized controlled trials (RCTs), prospective or retrospective cohorts or case-control studies; 2) reported on BM patients that underwent surgery or a subgroup that underwent surgery; 3) reported on risk factors for developing LMD. This study's outcome was risk factors for the development of LMD. Exclusion criteria were: 1) nonhuman studies, 2) primary brain tumors, 3) studies reporting on <10 LMD patients, 4) non-English publications.

### Data Extraction

Relevant data were extracted and grouped as follows: study characteristics including study design and sample size, patient characteristics, intracranial tumor characteristics, systemic cancer characteristics, treatment characteristics including previous surgery, chemotherapy, upfront radiotherapy schedule, and outcome measures including risk factors for LMD. The Newcastle-Ottawa scale (NOS) was used for cohort studies and case control studies to assess the risk of bias in all included studies.^11^

### Statistical Analysis

Data analysis was performed using R v 3.5.0 (R Core Team, Vienna, Austria). A meta-analysis was performed if more than three studies reported a specific risk factor with poolable effect size metrics. The random-effects model, combined with the DerSimonian-Laird method^12^ to account for variation between studies, was used to obtain the overall hazard ratio (HR) estimates and 95% confidence intervals (CI). If the standard error was not reported in the included studies, it was calculated using the HR and *P*-value.^13^ The meta-analysis was subsequently conducted using the metagen function of the meta package in R.^14^ The estimated results were visualized using forest plots. The Higgin's & Thompson's *I*^2^ was used to assess heterogeneity among studies^15^; >50% was considered high heterogeneity. A *P*-value < 0.1 for heterogeneity was considered significant.^16^

## Results

### Study Selection and Baseline Characteristics

**Figure 1.**
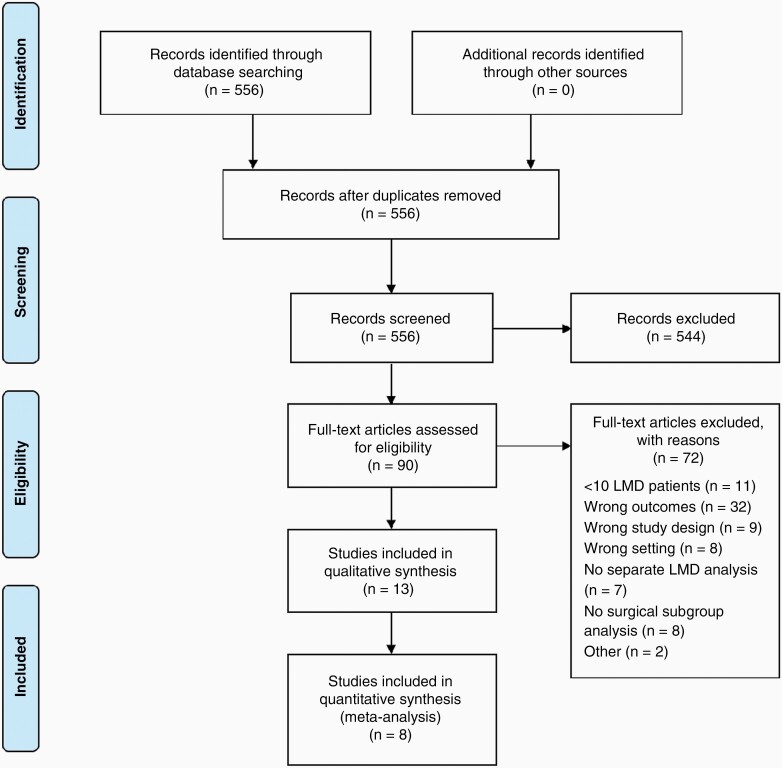
Flow diagram literature search.

After completing the search and removing duplicates, 556 publications were identified, of which thirteen studies met the inclusion criteria (Figure 1).^10,17–33^ No additional studies were identified by the reference check.

Included studies were case-control studies (*n* = 3),^26,28,30^ and retrospective cohorts (*n* = 10).^17–21,24,27,31–33^ The included studies described a total of 2105 patients, of which 386 (18.3%) developed LMD (Table 1). The median incidence of LMD across included studies was 16.1% with a median time to LMD from BM diagnosis of 6 months (3.8–14 months). The median follow-up time was 13.4 months in the included studies (8–16 months). LMD diagnosis was either defined by clinical presentation, CSF histology, or MRI (Table 1**).**

**Table 1.** Study Characteristics

Study	Study Duration	Country	Study Design (Single vs. Multicenter)^a^	Treatment Used	LMD Diagnosis^b^	Quality of Study (NOS)	Total Sample Size	Surgical Sample Size	Total Number of LMD Patients in surgically treated BM patients (%)	Median follow-up (in months)	Median time to LMD (range; in months)
Ahn et al (2012)^17^	2001–2009	Korea	Cohort (single)	R	CSF/MRI	8	242	242	39 (16.1)	8	6 (1.0–42.0)
Atalar et al. (2013)^18^	1998–2011	Turkey, USA	Cohort (single)	R + SRS	CSF/MRI	5	175	175	21 (12.0)	12.4	5 (2.0–33.0)
Ojerholm et al. (2014)^27^	2007–2013	-	Cohort (single)	R + gamma knife SRS	Clinic/CSF/MRI	8	91	91	12 (13.2)	15.3	–
Hsieh et al. (2015)^21^	2004–2012	USA	Cohort (single)	R + SRS/ WBRT/ IORT	ClinicalCSF	5	212	212	27 (12.7)	–	–
Patel et al. (2016)^28^	2005–2013	USA	Case-control (multi)	R + SRS before or after surgery	MRI	8	180	180	25 (13.9)	–	14 (NA)
Keller et al. (2017)^24^	2008–2015	France	Cohort (multi)	R + HSRT	Clinical/CSF/MRI	5	181	181	26 (14.4)	15.3	3.8 (0.13–33.6)
Press et al. (2019)^30^	2008–2017	USA	Case control (multi)	R + SRS; solitary BM	CSF/MRI	8	134	134	33 (24.6)	14.2	12.2 (1.2–52.3)
Foreman et al. (2018)^20^	2005–2015	USA	Cohort (single)	R + SRS/ HSRT	MRI	9	91	91	32 (35.2)	9.0	–
DePaoli et al. (2019)^19^	2009–2015	USA	Cohort (single)	R + SRS	CSF/MRI	9	50	50	12 (24.0)	12.9	6.0 (0.7–14.5)
Soliman et al. (2019)^31^	2009–2014	Canada	Cohort (single)	R + HSRT	MRI	8	122	122	32 (26.2)	16.0	–
Nguyen et al. (2020)^26^	–	Canada	Case-control (multi)	Intact BM treated with HSRT vs. R + HSRT	MRI	7	235	123	45 (36.6)	13.4	–
Shi et al. (2020)^33^	2007–2018	USA	Cohort (single)	R + SRS	MRI	7	442	442	70 (15.8)	10.1	–
Teyateeti et al. (2020)^32^	2013–2019	USA	Cohort (single)	R + single fraction SRS	MRI	7	62	62	12 (19.4)	15.1	–

^a^All included studies were retrospective studies.

^b^LMD diagnosis was either defined by clinical presentation, MRI, or cerebrospinal fluid histology.

LMD, leptomeningeal disease; BM, brain metastasis; CSF, cerebrospinal fluid; NOS, Newcastle-Ottawa Scale; SRS, stereotactic radiosurgery; HSRT, hypofractionated stereotactic radiotherapy; WBRT, whole brain radiation therapy; IORT, intraoperative radiation therapy; R, Resection.

Most studies reported exclusively on patients who underwent surgery for their newly diagnosed BM.^17–21,24,27,28,30–33^ One study reported on LMD risk in a broader newly diagnosed BM population consisting of surgical and nonsurgical patient subgroups.^26^ Only the surgical BM patients were included in this meta-analysis. The lung was the most reported primary tumor site (Table 2).

**Table 2.** Patient, Tumor, and Treatment Characteristics

Study	Primary Tumor Site (%)						Single BM (%)	GTR (%)	Adjuvant Radiation (%)	Neo-adjuvant Radiation (%)	Systemic Therapy (%)	Male Patients (%)	Median Age at Diagnosis
	Breast	Lung	Gastro-intestinal	Melanoma	Kidney	Other							
Ahn et al (2012)^17^	25 (10)	164 (68)	–	–	–	53 (22)	164 (68)	–	143 (59)	0	117 (48)	–	61
Atalar et al. (2013)^18^	27 (15)	76 (43)	18 (10)	24 (14)	–	30 (17)	–	160 (91)	165 (100)	0	24 (14)	67 (38)	60
Ojerholm et al. (2014)^27^	12 (13)	39 (43)	10 (11)	13 (14)	6 (7)	13 (12)	–	79 (82)	91 (100)	0	–	36 (40)	60
Hsieh et al. (2015)^21^	30 (14)	108 (51)	–	22 (10)	–	45 (22)	125 (59)	–	212 (100)	0	–	90 (42)	58
Patel et al. (2016)^28^	30 (16.7)	72 (40)	–	34 (18.8)	–	44 (24.4)	121 (67.2)	142 (78.9)	180 (100)	66 (36.7)	–	77 (40)	60
Keller et al. (2017)^24^	20 (11.1)	82 (45.3)	18 (9.9)	16 (8.8)	18 (9.9)	14 (15)	138 (73)	178 (94.2)	181 (100)	0	7 (3.8)	100 (55)	61
Press et al. (2019)^30^	21 (15.7)	64 (47.8)	–	25 (18.7)	–	24 (11.2)	38 (28.4)	96 (71.6)	134 (100)	0	30 (22.6)	55 (41)	59
Foreman et al. (2018)^20^	11 (12.1)	39 (42.9)	3 (3.3)	17 (18.7)	8 (8.8)	10 (14.3)	64 (70.3)	70 (76.9)	91 (100)	0	–	41 (45)	–
DePaoli et al. (2019)^19^	2 (4)	32 (64)	4 (8)	7 (14)	4 (8)	1 (2)	32 (64)	–	50 (100)	0	–	23 (46)	–
Soliman et al. (2019)^31^	25 (21)	56 (46)	7 (6)	9 (7)	10 (8)	15 (12)	89 (73)	122 (89)^a^	122 (100)^a^	0	37 (30)	43 (35)	–
Nguyen et al. (2020)^26^	30 (21.9)	60 (43.8)	11 (8.0)	9 (6.6)	11 (8.0)	16 (11.7)	90 (73.2)	123 (90.4)	123 (100)	0	38 (30.9)	–	62
Shi et al. (2020)^33^	78 (18)	52 (34)	53 (12)	50 (11)	27 (6)	82 (19)	–	447 (90)^b^	442 (100)^b^	75 (17)	269 (54)	214 (48)	62
Teyateeti et al. (2020)^32^	9 (15)	34 (55)	–	9 (15)	5 (8)	5 (8)	39 (63)	–	62 (100)	0	–	24 (39)	66

Abbreviations: GTR, gross total resection; BM, brain metastases.

^a^137 BM in total in 122 patients.

^b^501 BM in total in 442 patients.

Out of the 37 different possible risk factors mentioned in the studies, eighteen unique risk factors were reported to be significantly associated with the development of LMD by at least one study (Supplementary Table S2). One study^33^ identified risk factors for classical LMD and nodular LMD separately. The results of this study were summarized in this review but not included in the meta-analysis. The other studies grouped these two patterns together for their quantitative analysis.

### Patient Characteristic Risk Factors

Four patient characteristics (age,^17,19^ gender,^19^ Karnofsky performance status,^21^ and diagnosis-specific graded prognostic assessment (ds-GPA) score^21^) were analyzed as possible risk factors for LMD, but no significant association was demonstrated (Supplementary Table S2).

### Brain Tumor Characteristic Risk Factors

A total of seven brain tumor variables were reported for LMD: the tumor's largest dimension or volume,^19,24,32^ either involved CSF or contact to CSF,^17^ hemorrhagic features,^30^ cystic features,^30^ pial involvement,^18,32^ supra- versus infra-tentorial BM location,^18–20,24,27,32^ and number of BMs.^19–21,28,30,32^ Three tumor variables were identified as significant risk factors in multiple studies, namely number of BMs^19,28,30^ and proximity of BM to CSF,^17^ and supra- versus infra-tentorial location.^27,33^ Three variables were identified as significant in one study: BM size,^24^ and hemorrhagic or cystic features.^30^ Pial involvement was investigated in two studies but was significant in neither^18,32^ (Table 3).

**Table 3.** Risk Factors Significantly Associated With Time-to-leptomeningeal Disease per Category

	Risk Factor	Number of Studies Reporting Risk Factor	Study	HR [95% CI]; *P*-value	Uni- or Multi-variate	Corrected for Number of BM	Corrected for Primary Tumor
Brain tumor characteristics	Number of metastases	7	DePaoli (2019)^19^ Press (2019)^30^ Patel (2016)^28^ Shi (2020)^,33^^a^	HR = 1.92 [0.84–2.11]; *P* = .03 HR = 3.23 [1.20–8.40]; *P* = .02 HR = 1.35 [1.15–1.60]; *P* < .001 HR = 0.39 [0.19–0.79]; *P* = .01	Multivariate Multivariate Multivariate Univariate	Yes – Yes –	No Yes Yes –
	Tentorial location of BM (infratentorial vs. supratentorial)	7	Ojerholm (2014)^27^ Shi (2020)^33,^^c^	HR = 4.60 [1.40–14.90]; *P* = .01 HR = 2.18 [1.16–4.08]; *P* = .01	Multivariate Univariate	Yes –	Yes –
	Presurgical tumor volume	6	Keller (2017)^24^	HR = 1.02 [1.0–1.04]; p = 0.03	Univariate	–	–
	Contact to CSF	2	Ahn (2012)^17,^^d^	HR = 6.31 [1.31–30.38]; *P* = .02	Multivariate	No	Yes
	Involved CSF	2	Ahn (2012)^17,^^e^	HR = 9.0 [2.12–38.27]; *P* < .01	Multivariate	No	Yes
	Hemorrhagic features	1	Press (2019)^30,^^f^	HR = 2.30 [0.79–1.95]; *P* = .04	Multivariate	Yes	Yes
	Cystic features	2	Press (2019)^30,^^g^	HR = 2.02 [0.86–2.13]; *P* = .02	Multivariate	Yes	Yes
Systemic cancer characteristics	Breast cancer as primary tumor type	7	Ojerholm (2014)^27^	HR = 3.80 [1.20–12.4]; *P* = .02	Multivariate	Yes	–
	HER2 receptor	4	Press (2019)^30^	HR = 0.15 [0.24–0.79]; *P* = .02	Multivariate	Yes	Yes
Treatment characteristics	Extent of resection (STR vs. GTR)	3	Soliman (2019)^31,^^h^	HR = 2.90 [1.40–5.90]; *P* = .01	Multivariate	–	Yes
	Method of resection (piecemeal vs. en bloc)	4	Ahn (2012)^17,^^h^	HR = 3.67 [1.22–11.0]; *P* = .02	Multivariate	No	Yes
	Ventricle violation during surgery	1	DePaoli (2019)^19,^^i^	HR = 7.12 [0.61–9.06]; *P* = .03	Multivariate	Yes	No
	Intracranial failure^b^	1	Teyateeti (2020)^32^	HR = 5.11 [1.52–17.22]; *P* = .003	Univariate	Yes	Yes
	Targeted therapy within 3 months of SRS	2	Shi (2020)^33,^^c^	HR = 1.97 [1.009–3.86]; *P* = .047	Univariate	–	–
	Hormonal therapy within 3 months of SRS	2	Shi (2020)^33,^^b^	HR = 2.96 [1.16–7.56]; *P* = .02	Univariate	–	–
	Type of radiation (local radiotherapy vs. WBRT)	2	Hsieh (2015)^21^	HR = 2.45 [1.03–5.83]; *P* = .04	Multivariate	Yes	No
	Year of SRS treatment	1	Shi (2020)^33,^^c^	HR = 1.25 [1.14–1.37]; *P* < .0001	Univariate	–	–
	Postoperative SRS vs. preoperative SRS	2	Nguyen (2020)^26^ Patel (2016)^28^	HR = 2.12 [0.90–1.72]; *P* = .01 HR = 4.03 [1.20–13.60]; *P* = .02	Multivariate Multivariate	Yes Yes	Yes Yes

Abbreviations: HR, *h*azard ratio; BM, brain metastases; CSF, cerebrospinal fluid; HER2, Human epidermal growth factor receptor 2; STR, subtotal resection; GTR, gross total resection; WBRT, whole brain radiation therapy; SRS, stereotactic radiosurgery.

^a^Associated with nodular LMD (this study distinguished between nodular and classical LMD in the analyses).

^b^Defined as any residual tumor or other BM. Residual tumor was defined by the method of Susko et al.^30^with recurrences defined as either in-field (within the planning target volume (PTV)) or marginal (outside the PTV but within the volume defined by 50% of the prescription dose).

^c^Associated with classical LMD (this study distinguished between nodular and classical LMD in the analyses).

^d^Contact to CSF was defined as the surface of the tumor in contact with the pia mater or the ventricle wall without intervening brain parenchyma.

^e^Involved CSF was defined as pial or ependymal enhancement or asymmetrical cortical vessel enhancement accompanied by the criteria of “contact” location.

^f^Hemorrhagic features were defined as lesions on MRI with hyperintensity on T1-, and/or hypointensity on T2-weighted imaging, as well as hypodensities on noncontrasted CT.

^g^Cystic features were defined as lesions containing discrete fluid-filled components which were hyperintense on T1- and hyperintense on T2-weighted imaging.

^h^No detailed definition was given for this variable.

^i^Ventricle violation was defined as the surgical field entering into the ventricles as described in the operative notes and on postoperative T2-weighed imaging.

### Systemic Cancer Characteristic Risk Factors

Nine systemic cancer characteristics were identified in the current literature (Supplementary Table S2): time from primary cancer to BM,^17^ stable primary tumor,^26^ breast cancer as primary tumor type,^17,18,20,27,30,31^ melanoma as primary tumor type,^31,32^ nonsmall cell lung cancer as primary tumor type,^17,32^ small cell lung cancer as primary tumor type,^17^ unspecified lung cancer as primary tumor type,^33^ renal cell carcinoma as primary tumor type,^33^ and the molecular subtype of breast cancer.^17,18,30^ Breast cancer as the primary tumor type significantly increased the risk of LMD in four studies after using the estimated SE in the random-effects model.^20,27,30,31^ HER2-receptor sensitivity decreased the risk of LMD in one study^30^ (Table 3).

### Treatment Characteristic Risk Factors

A total of seventeen treatment variables were reported for LMD: type of systemic therapy,^30^ extent of resection,^20,31^ method of resection,^17,18,24,30^ aspiration of BM,^30^ use of cavitron ultrasonic surgical aspirator (CUSA),^17^ ventricle violation during surgery,^19^ prior therapy of BM,^33^ surgical cavity control,^18^ intracranial failure defined as any residual tumor or other BM after resection,^32^ time from surgery to SRS,^19,20,32^ gamma knife versus linear accelerator (LINAC) radiosurgery,^20^ local radiotherapy versus whole brain radiotherapy (WBRT),^21^ radiation dose,^32^ sensitivity to hypofractionated radiation,^26^ single fraction versus multi-fraction SRS,^33^ preoperative versus postoperative SRS,^26,28^ and the year of SRS receipt.^33^ In total, nine treatment variables were significantly associated with increased LMD risk; Postoperative versus preoperative SRS was significantly increased LMD risk in two studies,^26,28^ while hormonal therapy within three months of adjuvant SRS,^33^ targeted therapy within three months of adjuvant SRS,^33^ subtotal resection,^31^ piecemeal resection,^17^ ventricle violation during surgery,^19^ intracranial failure,^32^ and local radiation versus WBRT^21^ were reported to increase the LMD risk significantly in one study. The year of SRS receipt was significantly associated with increased classical LMD risk specifically.^33^

### Meta-analysis of LMD Risk Factors

**Figure 2.**
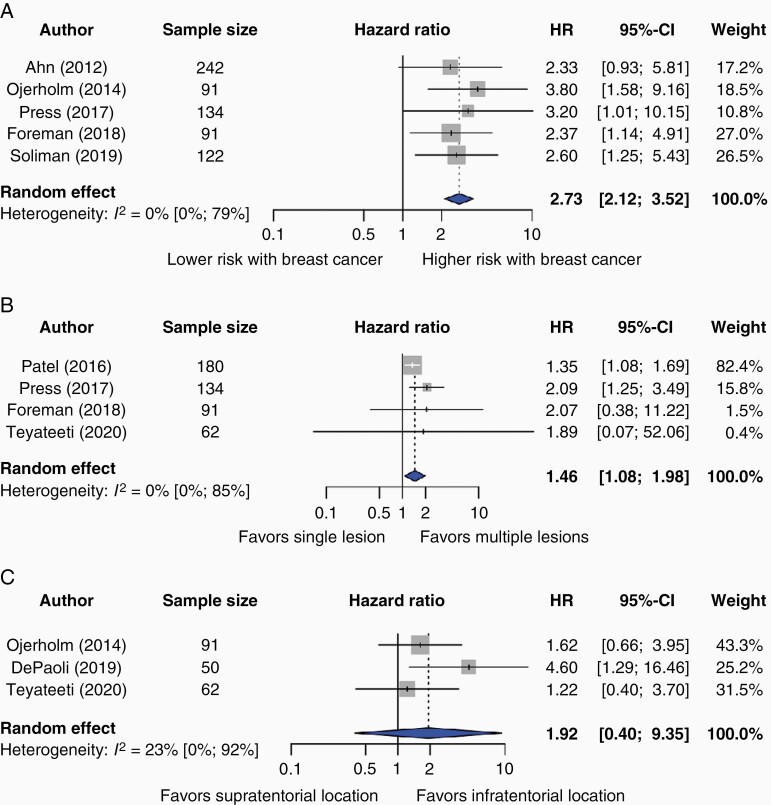
Forest plot of hazard ratios (HR) and 95% confidence intervals (CI) of the risk of leptomeningeal disease (LMD) by (A) breast cancer as primary tumor type, (B) multiple brain lesions, and (C) tentorial location of BM. The gray squares represent the point estimate of each study; the size of the squares is proportional to the weight of the study; horizontal lines show the 95% confidence intervals (CIs); the center of the blue diamond represents the pooled estimate for each category. A) The pooled hazard ratio (HR) for the risk factor breast cancer as primary tumor type is 2.73 (95% CI 2.12–3.52; *I*^2^ = 0%, p-heterogeneity = 0.93; 5 studies). B) The pooled HR for the risk factor multiple brain lesions is 1.37 (95% CI 1.18–1.58; *I*^2^ = 0%, p-heterogeneity = 0.92; 4 studies). C) The pooled HR for the risk factor tentorial location of BM is 2.24 (95% CI 0.36–13.75; *I*^2^ = 48%, p-heterogeneity = 0.15; 3 studies). A *P*-value for heterogeneity <10% was considered significant.

Three unique risk factors were reported consistently by more than three studies, enabling meta-analysis. Breast cancer origin (HR 2.73, 95% CI: 2.12–3.52; five studies^17,20,27,30,31^) and multiple BMs (HR 1.37, 95% CI: 1.18–1.58; four studies^20,28,30,32^) were associated with a higher risk of LMD; Figure 2A and B). No significant heterogeneity was observed in these analyses (*I*^2^ = 0% for both; p-heterogeneity = 0.93 and p-heterogeneity = 0.92 for breast cancer and multiple BMs, respectively). Infratentorial BM location was consistently reported by three studies,^19,27,32^ but was not significantly associated with the occurrence of LMD (HR 2.24, 95% CI: 0.36–13.75, *I*^2^ = 48%, p-heterogeneity = 0.15; Figure 2C). Three studies eligible for meta-analysis reported time from surgery to SRS; However, two studies^20,32^ defined this variable in a dichotomous manner, eg less than eleven days versus more than eleven days. One study defined time from surgery to SRS as a numerical variable.^19^ Therefore, meta-analysis for this variable was not possible.

## Discussion

This systematic review and meta-analysis were performed to summarize the LMD risk factors. At least one study reported the number of BMs, primary tumor site, proximity of BM to the CSF, hemorrhagic and cystic tumor features, and tumor size to increase the risk of LMD. Positive HER-2 receptor status in breast cancer BM patients was reported to decrease LMD development risk. Pooling effect sizes demonstrated that breast cancer as the primary tumor site and multiple BMs were significantly associated with a higher risk of LMD. No significant association was demonstrated between an infratentorial location or tumor size and a higher risk of LMD.

Metastatic breast cancer recurs as an essential risk factor for LMD irrespective of treatment modality.^18,23,34,35^ The hypothesis for metastatic breast cancer as a risk factor is that a specific tropism in tumor subtype, ie hormonal receptor status and HER2-status, could potentiate metastatic breast cancer to more likely settle in the leptomeninges than other primary tumor locations.^35^ This is, however, contradictory to current research in a broader breast cancer patient population where positive HER2-status was associated with improved survival after LMD diagnosis.^36^ No association was observed between estrogen and progesterone receptor status.^18^

The pooled results of four studies demonstrated multiple BMs to be associated with a higher LMD risk. However, one study that could not be included in the meta-analysis due to separate analyses for classical and nodular LMD demonstrated a lower nodular LMD risk in patients with multiple BMs.^33^ This observation is inconsistent with the current literature and could be a spurious finding in their univariate analysis.

This paper specifically aimed to identify risk factors for LMD within neurosurgical patients. However, another open question is whether surgery itself increases the risk of LMD. The literature demonstrated contrasting results regarding the increased risk of LMD in neurosurgical treated BM patients compared with SRS.^9,10,23,25,29,37,38^ Five studies reported that prior neurosurgical resection was significantly associated with an increased LMD risk compared to SRS.^9,23,25,37,38^ However, two studies reported this significant association only for piecemeal resection compared to SRS; ^10,29^ increased LMD risk was not observed for en bloc BM resection. Only one included^17^ study reported a significant difference in LMD risk when comparing piecemeal versus en bloc resection. In the current literature, en bloc resection had an LMD risk comparable to SRS.^10,29^ In other studies, neurosurgical patients with no further specification of the method of resection had an increased risk of LMD compared with BM patients receiving only SRS.^9,23,25,37^ The suggestion was made by Suki et al.^10^ to always strive for en bloc resection. Pragmatically, this is not possible; the firmness of capsules between BMs differs empirically by primary tumor type, which makes en bloc resection not always possible. Additionally, BMs adjacent to eloquent brain tissue can complicate performing an en bloc resection, as the BM needs to be dissected from critical neurologic structures resulting in neurologic deficits. This dilemma must be considered when the neurosurgeon and patient decide on the BM treatment. This creates confounding by indication; piecemeal resections are more likely to recur and increase LMD risk. Only two^24,30^ of the five studies^17,18,23,24,30^ discussing the method of resection corrected for the extent of resection.

Moreover, the treatment sequence might be relevant in LMD development; Two studies reported a significantly decreased LMD risk for SRS on intact BMs versus cavity SRS,^26,28^ whereas one study found no significant association.^39^ Preoperative SRS might restrict tumor cell dissemination during surgery hypothetically by sterilizing the treatment field before surgery, explaining the observed findings.^40^ Currently, sequencing radiation therapy before surgical resection is further being researched. Presurgical radiation therapy could reduce the LMD risk but comes with its own set of risks for surgical resection, such as worsened wound healing.^41^

The development of LMD is most known to be caused by CSF seeding through the leptomeninges by hematogenic, perineural, or direct BM expansion.^17^ The latter is likelier to happen when BMs have direct contact with CSF-producing or -carrying structures.^42,43^

The manipulation of CSF structures concerning BM location or intraoperative ventricle violation was also a recurring LMD risk factor in the included studies.^17,19^ While these risk factors were not poolable, both studies reported high effect sizes for increased LMD risk.

Potential LMD risk factors, such as infratentorial BM location and large BM size, are empirically associated with LMD occurrence in the neurosurgical clinic. However, these associations were not reflected in this analysis; Tumor size was only associated with increased LMD risk in one^24^ of the three studies reporting on it and did not correct for surgical covariates, which are hypothesized to cause LMD by tumor spillage. Infratentorial BM location was also not significantly associated with increased LMD risk; only one^27^ of the six studies reporting on it observed a significant association. Two multivariate analyses^20,27^ corrected for the extent of resection and all studies corrected for BM size and number of BMs. Both confounders were reported to be associated with an increased LMD risk.^19,24,28,30^ Moreover, only two studies^20,27^ corrected for primary tumor location, ie breast cancer BMs versus other metastases. Sample sizes of all studies mentioned above were limited, ranging from 50 to 181 with even fewer LMD cases. This could restrain the generalizability and re-emphasizes the need for larger sample sizes in future studies.

This study had several limitations. First, the heterogeneity of (neo-)adjuvant treatment modalities used in the included studies prevents us from ascertaining specific and conclusive recommendations. However, all analyzed study (sub-)groups underwent a resection for their primary BM. Second, relatively few variables could be used for the meta-analysis due to the lack of consistent reporting of effect sizes. Unfortunately, we were unable to retrieve the missing data for the meta-analysis by contacting the corresponding authors. Furthermore, some variables were reported in different studies using noninterchangeable effect sizes, eg relative risk and hazard ratio, which prevented pooling of the results. The uniformity of extracted variables between included studies also differed greatly, which reduced the possible number of meta-analyses.

Third, a possibly skewed representation of risk factors of LMD is shown as some included studies only reported on statistically significant LMD risk factors.^25,31^ To avoid biasing our representation towards positive results, we reported how many studies identified a given variable as a significant risk factor and how many investigated this variable and did not find it significant. Fourth, the included study duration varied from 1998 until 2019; However, the evolution of therapeutic options and the improvement of diagnostic tools may have influenced the course of disease after surgery without or with radiotherapy for brain metastasis. This hypothesis is supported by the significant association of year of performed SRS treatment and increased classical LMD risk in one included study.^33^

Our study's major strength is that it was the first meta-analysis that focused on the risk of LMD occurrence for surgically treated BM patients. Moreover, the majority of included studies used multivariate analysis, which decreased confounding bias.

Future research should investigate the relation of different treatment modalities and the use of surgical instruments and LMD. Studies should investigate potential risk factors that can be mitigated, such as the treatment sequence and choice of surgical technique. Prognostic models for LMD should also be investigated to understand LMD occurrence further. Furthermore, prospective randomized studies should be performed regarding the effect of different treatment modalities on LMD risk and the optimal timing of (adjuvant) treatment(s) to LMD. Finally, more preclinical research should be conducted in BM models to explain the high LMD risk in primary breast cancer patients.

## Conclusion

Breast cancer as the primary tumor location and multiple BMs increase the risk of LMD occurrence. Important neurosurgical risk factors, including the proximity of BM to CSF structures, ventricle violation during surgery, and the method of resection might influence the occurrence of LMD. Further research should focus on the effect of different treatment modalities on LMD risk, as well as the optimal sequence of treatments.

## Supplementary Material

vdab162_suppl_Supplementary_Materials
