# Supplementary material for: Leptomeningeal disease in neurosurgical brain metastases patients: A systematic review and meta-analysis
Source: Neurooncol Adv. 2021 Nov 10;3(1):vdab162. doi: 10.1093/noajnl/vdab162 (PMC8633671; doi:10.1093/noajnl/vdab162)
Supplement: vdab162_suppl_Supplementary_Materials [file vdab162_suppl_supplementary_materials.docx]

Supplementary material 1

**PubMed**

<http://www.ncbi.nlm.nih.gov/pubmed?otool=leiden>

(("Meningeal Carcinomatosis"[Mesh] OR meningeal carcinomat*[tw] OR carcinomatous meningit*[tw] OR leptomeningeal carcinomatos*[tw] OR leptomeningeal disease*[tw] OR leptomeningeal seed*[tw] OR "Neoplasm Seeding"[Mesh] OR neoplasm seeding[tw] OR "Tumor Seeding"[tw] OR "Tumour Seeding"[tw] OR leptomeningeal recurrence*[tw] OR leptomening*[tw] OR lepto-mening*[tw]) AND (brain metastas*[tw] OR cerebral metastas*[tw] OR (("Brain Neoplasms"[mesh:noexp] OR brain neoplasm*[tw] OR brain tumor*[tw] OR brain tumour*[tw] OR brain cancer*[tw] OR cancer of brain*[tw] OR cancer of the brain*[tw] OR intracranial neoplasm*[tw] OR cerebral neoplasm*[tw] OR cerebral tumor*[tw] OR cerebral tumour*[tw] OR cerebral cancer*[tw] OR central nervous system [tw] OR CNS [tw] OR cerebellar [tw] OR cranial [tw] OR skull [tw] OR pituitary [tw] OR "brain"[tw]) AND ("Neoplasm Metastasis"[mesh] OR metastas*[tw] OR metastat*[tw]))) AND ("Craniotomy"[Mesh] OR craniotom*[tw] OR "Stereotaxic Techniques"[Mesh] OR stereotaxic techni*[tw] OR stereotactic techni*[tw] OR stereota*[tw] OR "surgery"[Subheading] OR surger*[tw] OR surgical*[tw] OR resection*[tw] OR resected[tw] OR neurosurg*[tw]) NOT ("Animals"[mesh] NOT "Humans"[mesh]) NOT (("Case Reports"[ptyp] OR "case report"[ti] OR "Review"[ptyp] OR "review"[ti]) NOT ("Clinical Study"[ptyp] OR "trial"[ti] OR "RCT"[ti] OR "case series"[ti])))

**Embase**

<http://ovidsp.ovid.com/ovidweb.cgi?T=JS&PAGE=main&MODE=ovid&D=oemezd>

((*"Carcinomatosis Meningitis"/ OR "meningeal carcinomat*".ti,ab OR "carcinomatous meningit*".ti,ab OR "leptomeningeal carcinomatos*".ti,ab OR "leptomeningeal disease*".ti,ab OR "leptomeningeal seed*".ti,ab OR *"Tumor Seeding"/ OR "neoplasm seeding".ti,ab OR "tumor seeding".ti,ab OR "tumour seeding".ti,ab OR "leptomeningeal recurrence*".ti,ab OR leptomening*.ti,ab OR "lepto-mening*".ti,ab) AND (*"Brain Metastasis"/ OR "brain metastas*".ti,ab OR "cerebral metastas*".ti,ab OR ((*"Brain Tumor"/ OR "brain neoplasm*".ti,ab OR "brain tumor*".ti,ab OR "brain tumour*".ti,ab OR "brain cancer*".ti,ab OR "cancer of brain*".ti,ab OR "cancer of the brain*".ti,ab OR "intracranial neoplasm*".ti,ab OR "cerebral neoplasm*".ti,ab OR "cerebral tumor*".ti,ab OR "cerebral tumour*".ti,ab OR "cerebral cancer*".ti,ab OR "central nervous system".ti,ab OR "CNS".ti,ab OR "cerebellar".ti,ab OR "cranial".ti,ab OR "skull".ti,ab OR "pituitary".ti,ab OR "brain".ti,ab) AND (exp *"Metastasis"/ OR metastas*.ti,ab OR metastat*.ti,ab))) AND (*"Craniotomy"/ OR craniotom*.ti,ab OR exp *"Stereotactic Procedure"/ OR "stereotaxic techni*".ti,ab OR "stereotactic techni*".ti,ab OR stereota*.ti,ab OR surger*.ti,ab OR surgical*.ti,ab OR resection*.ti,ab OR "resected".ti,ab OR neurosurg*.ti,ab) NOT (exp "Animals"/ NOT exp "Humans"/) NOT (("Case Report"/ OR "case report".ti OR "Review"/ OR "review".ti) NOT ("Clinical Study"/ OR "trial".ti OR "RCT".ti OR "case series".ti OR exp "Clinical Trial"/))) NOT (conference review or conference abstract).pt

**Web of Science**

<http://isiknowledge.com/wos>

(TI=("Carcinomatosis Meningitis" OR "meningeal carcinomat*" OR "carcinomatous meningit*" OR "leptomeningeal carcinomatos*" OR "leptomeningeal disease*" OR "leptomeningeal seed*" OR "Tumor Seeding" OR "neoplasm seeding" OR "tumor seeding" OR "tumour seeding" OR "leptomeningeal recurrence*" OR leptomening* OR "lepto-mening*") AND TS=("Brain Metastasis" OR "brain metastas*" OR "cerebral metastas*" OR (("Brain Tumor" OR "brain neoplasm*" OR "brain tumor*" OR "brain tumour*" OR "brain cancer*" OR "cancer of brain*" OR "cancer of the brain*" OR "intracranial neoplasm*" OR "cerebral neoplasm*" OR "cerebral tumor*" OR "cerebral tumour*" OR "cerebral cancer*" OR "central nervous system" OR "CNS" OR "cerebellar" OR "cranial" OR "skull" OR "pituitary" OR "brain") AND ("Metastasis" OR metastas* OR metastat*))) AND TS=("Craniotomy" OR craniotom* OR "Stereotactic Procedure" OR "stereotaxic techni*" OR "stereotactic techni*" OR stereota* OR surger* OR surgical* OR resection* OR "resected" OR neurosurg*) NOT ti=(("Case Report" OR "case report" OR "Review" OR "review") NOT ("Clinical Study" OR "trial" OR "RCT" OR "case series" OR "Clinical Trial")) **NOT ti=("veterinary" OR "rabbit" OR "rabbits" OR "animal" OR "animals" OR "mouse" OR "mice" OR "rodent" OR "rodents" OR "rat" OR "rats" OR "pig" OR "pigs" OR "porcine" OR "horse" OR "horses" OR "equine" OR "cow" OR "cows" OR "bovine" OR "goat" OR "goats" OR "sheep" OR "ovine" OR "canine" OR "dog" OR "dogs" OR "feline" OR "cat" OR "cats") NOT dt=(meeting abstract)) OR** (TS=("Carcinomatosis Meningitis" OR "meningeal carcinomat*" OR "carcinomatous meningit*" OR "leptomeningeal carcinomatos*" OR "leptomeningeal disease*" OR "leptomeningeal seed*" OR "Tumor Seeding" OR "neoplasm seeding" OR "tumor seeding" OR "tumour seeding" OR "leptomeningeal recurrence*" OR leptomening* OR "lepto-mening*") AND TI=("Brain Metastasis" OR "brain metastas*" OR "cerebral metastas*" OR (("Brain Tumor" OR "brain neoplasm*" OR "brain tumor*" OR "brain tumour*" OR "brain cancer*" OR "cancer of brain*" OR "cancer of the brain*" OR "intracranial neoplasm*" OR "cerebral neoplasm*" OR "cerebral tumor*" OR "cerebral tumour*" OR "cerebral cancer*" OR "central nervous system" OR "CNS" OR "cerebellar" OR "cranial" OR "skull" OR "pituitary" OR "brain") AND ("Metastasis" OR metastas* OR metastat*))) AND TS=("Craniotomy" OR craniotom* OR "Stereotactic Procedure" OR "stereotaxic techni*" OR "stereotactic techni*" OR stereota* OR surger* OR surgical* OR resection* OR "resected" OR neurosurg*) NOT ti=(("Case Report" OR "case report" OR "Review" OR "review") NOT ("Clinical Study" OR "trial" OR "RCT" OR "case series" OR "Clinical Trial")) **NOT ti=("veterinary" OR "rabbit" OR "rabbits" OR "animal" OR "animals" OR "mouse" OR "mice" OR "rodent" OR "rodents" OR "rat" OR "rats" OR "pig" OR "pigs" OR "porcine" OR "horse" OR "horses" OR "equine" OR "cow" OR "cows" OR "bovine" OR "goat" OR "goats" OR "sheep" OR "ovine" OR "canine" OR "dog" OR "dogs" OR "feline" OR "cat" OR "cats") NOT dt=(meeting abstract))**

**Cochrane**

<https://www.cochranelibrary.com/advanced-search/search-manager>

(("Carcinomatosis Meningitis" OR "meningeal carcinomatosis" OR "carcinomatous meningitis" OR "leptomeningeal carcinomatos*" OR "leptomeningeal disease" OR "leptomeningeal seeding" OR "Tumor Seeding" OR "neoplasm seeding" OR "tumor seeding" OR "tumour seeding" OR "leptomeningeal recurrence" OR leptomeningitis OR "lepto meningitis"):ti,ab,kw AND ("Brain Metastasis" OR "brain metastastatic" OR "cerebral metastasis" OR (("Brain Tumor" OR "brain neoplasm*" OR "brain tumors" OR "brain tumour" OR "brain cancer" OR "cancer of brain" OR "cancer of the brain" OR "intracranial neoplasm" OR "cerebral neoplasm" OR "cerebral tumor" OR "cerebral tumour" OR "cerebral cancer" OR "central nervous system" OR "CNS" OR "cerebellar" OR "cranial" OR "skull" OR "pituitary" OR "brain") AND ("Metastasis" OR metastastic OR metastat*))):ti,ab,kw AND ("Craniotomy" OR craniotomic OR "Stereotactic Procedure" OR "stereotaxic" OR stereota* OR surgery OR surgical OR resection OR "resected" OR neurosurgery):ti,ab,kw)

**NOT dt=(meeting abstract)**

**Emcare** <http://ovidsp.ovid.com/ovidweb.cgi?T=JS&NEWS=n&CSC=Y&PAGE=main&D=emcr>

((*"Carcinomatosis Meningitis"/ OR "meningeal carcinomat*".ti,ab OR "carcinomatous meningit*".ti,ab OR "leptomeningeal carcinomatos*".ti,ab OR "leptomeningeal disease*".ti,ab OR "leptomeningeal seed*".ti,ab OR *"Tumor Seeding"/ OR "neoplasm seeding".ti,ab OR "tumor seeding".ti,ab OR "tumour seeding".ti,ab OR "leptomeningeal recurrence*".ti,ab OR leptomening*.ti,ab OR "lepto-mening*".ti,ab) AND (*"Brain Metastasis"/ OR "brain metastas*".ti,ab OR "cerebral metastas*".ti,ab OR ((*"Brain Tumor"/ OR "brain neoplasm*".ti,ab OR "brain tumor*".ti,ab OR "brain tumour*".ti,ab OR "brain cancer*".ti,ab OR "cancer of brain*".ti,ab OR "cancer of the brain*".ti,ab OR "intracranial neoplasm*".ti,ab OR "cerebral neoplasm*".ti,ab OR "cerebral tumor*".ti,ab OR "cerebral tumour*".ti,ab OR "cerebral cancer*".ti,ab OR "central nervous system".ti,ab OR "CNS".ti,ab OR "cerebellar".ti,ab OR "cranial".ti,ab OR "skull".ti,ab OR "pituitary".ti,ab OR "brain".ti,ab) AND (exp *"Metastasis"/ OR metastas*.ti,ab OR metastat*.ti,ab))) AND (*"Craniotomy"/ OR craniotom*.ti,ab OR exp *"Stereotactic Procedure"/ OR "stereotaxic techni*".ti,ab OR "stereotactic techni*".ti,ab OR stereota*.ti,ab OR surger*.ti,ab OR surgical*.ti,ab OR resection*.ti,ab OR "resected".ti,ab OR neurosurg*.ti,ab) NOT (exp "Animals"/ NOT exp "Humans"/) NOT (("Case Report"/ OR "case report".ti OR "Review"/ OR "review".ti) NOT ("Clinical Study"/ OR "trial".ti OR "RCT".ti OR "case series".ti OR exp "Clinical Trial"/)))

| **Table S2. Risk factors for leptomeningeal disease per category** | | | |
| --- | --- | --- | --- |
|  | **Risk factor** | **Number of studies reporting on risk factors** | **Number of studies reporting this risk factor to be significant (n = 23)** |
| **Patients’ characteristics** | Age | 3 | 0 |
|  | Gender | 2 | 0 |
|  | KPS | 1 | 0 |
|  | GPA | 1 | 0 |
| **Brain tumor characteristics** | Number of BM | 7 | 4 |
|  | Tentorial location of BM (infratentorial vs. supratentorial) | 7 | 2 |
|  | Tumor’s largest dimension or volume | 6 | 1 |
|  | Proximity of BM to CSF (contact, involved) | 2 | 2 |
|  | Pial involvement | 3 | 0 |
|  | Hemorrhagic features | 1 | 1 |
|  | Cystic features | 2 | 1 |
| **Systemic cancer characteristics** | Time from diagnosis of primary cancer to BM | 2 | 0 |
|  | Stable primary tumor | 1 | 1 |
|  | Breast cancer as primary tumor type | 7 | 1 |
|  | Melanoma as primary tumor type | 3 | 0 |
|  | NSCLC as primary tumor type | 2 | 0 |
|  | SCLC as primary tumor type | 1 | 0 |
|  | Lung cancer as primary tumor type | 1 | 0 |
|  | RCC as primary tumor type | 1 | 0 |
|  | Molecular subtype breast cancer (HER2, PR, ER) | 4 | 1 |
| **Treatment characteristics** | Type of systemic therapy^1^ | 2 | 1 |
|  | Extent of resection (STR vs. GTR) | 3 | 1 |
|  | Method of resection (piecemeal vs. en bloc) | 4 | 1 |
|  | Aspiration of BM^2^ | 1 | 0 |
|  | Use of CUSA | 1 | 0 |
|  | Ventricle violation during surgery | 1 | 1 |
|  | Cavity local control | 1 | 0 |
|  | Intracranial failure^3^ | 1 | 1 |
|  | Time from surgery to SRS | 4 | 0 |
|  | Prior therapy^4^ | 1 | 0 |
|  | Year of SRS treatment | 1 | 1 |
|  | Type of radiation therapy   - Gamma knife versus linear accelerator radiosurgery - Local radiotherapy versus WBRT | 1  1 | 0  1 |
|  | Radiation dose | 2 | 0 |
|  | Single fraction vs. multi-fraction SRS | 1 | 0 |
|  | Sensitivity to HSRT^5^ | 1 | 0 |
|  | Preoperative versus postoperative SRS | 2 | 2 |
| ^1^ Chemotherapy, targeted therapy or immunotherapy  ^2^ A BM was considered to be aspirated if lesion associated fluid was removed prior to resection  ^3^ Defined as any residual tumor or other BM  ^4^ Prior surgery or SRS to other BM, or to target lesion; prior whole brain radiation therapy  ^5^ Defined as radiosensitive versus radioresistant BMs based on tumor histology  *KPS* Karnofsky Performance Score, *GPA* Graded prognostic assessment, *BM* brain metastases, *CSF* cerebrospinal fluid, *NSCLC* non-small cell lung cancer, *SCLC* small cell lung cancer, *RCC* renal cell carcinoma, *HER2* Human epidermal growth factor receptor 2, *PR* Progesterone receptor, *ER* Estrogen receptor, *STR* subtotal resection, *GTR* gross total resection, *CUSA* Cavitron ultrasonic surgical aspirator, *SRS* stereotactic radiosurgery, *HSRT* hypofractionated stereotactic radiotherapy | | | |
